# Differentially expressed genes in head kidney of *Pelteobagrus fulvidraco* following *Vibrio cholerae* challenge

**DOI:** 10.3389/fimmu.2022.1039956

**Published:** 2023-01-10

**Authors:** Sen-Hao Jiang, Lin-Xin Wu, Yu-Ting Cai, Rui-Ting Ma, Hua-Bin Zhang, Dai-Zhen Zhang, Bo-Ping Tang, Qiu-Ning Liu, Li-Shang Dai

**Affiliations:** ^1^ Jiangsu Key Laboratory for Bioresources of Saline Soils, Jiangsu Synthetic Innovation Center for Coastal Bio-agriculture, Jiangsu Provincial Key Laboratory of Coastal Wetland Bioresources and Environmental Protection, School of Wetlands, Yancheng Teachers University, Yancheng, China; ^2^ School of Pharmaceutical Sciences, Wenzhou Medical University, Wenzhou, China; ^3^ School of Urban and Planning, Yancheng Teachers University, Yancheng, China

**Keywords:** pelteobagrus fulvidraco, transcriptome, *Vibrio cholerae*, differential expressed genes, immune response

## Abstract

The yellow catfish (*Pelteobagrus fulvidraco*) is a freshwater fish with high economic value in eastern China. Nevertheless, pathogens causing bacterial diseases in *P. fulvidraco* have brought about huge economic loss and high mortality in artificial aquaculture. For disease control, it is critical to further understand the immune system of yellow catfish and immune-related genes with which they respond to pathogenic infections. In this study, high-throughput sequencing methods were used to analyze the transcriptomic spectrum of the head kidney from *P. fulvidraco* challenged by *Vibrio cholera*. A total of 45,544 unique transcript fragments (unigenes) were acquired after assembly and annotation, with an average length of 1,373 bp. Additionally, 674 differentially expressed genes (DEGs) were identified after stimulation with *V. cholerae*, 353 and 321 genes were identified as remarkably up- or downregulated, respectively. To further study the immune-related DEGs, we performed KEGG enrichment and GO enrichment. The results showed gene regulation of response to stimulus, immune response, immune system progress, response to external stimuli and cellular response to stimuli. Analysis of KEGG enrichment is important to identify chief immune related pathways. Real-time quantitative reverse transcription-PCR (qRT-PCR) results indicated 10 immune response genes that were found to be upregulated compared to a control group after 6 h of *V. cholerae* challenging. In summary, the results of our study are helpful to determine the defense mechanisms and immune system responses of yellow catfish in reaction to bacterial challenges.

## Introduction

Yellow catfish has been extensively farmed and is an economically significant freshwater species in China, South Korea, Japan, and South Asia (1–3). The popularity of this species has rapidly increased recently because of its tender flesh, few bones, and good taste (4, 5). Consequently, the market demand for yellow catfish in China is very large, and its price very high. In recent years, the rapid growth of *P. fulvidraco* aquaculture has caused a huge decline in their population due to microbial disease, including viruses, parasites, and bacteria, which has brought about huge economic losses (6). However, little is known about the molecular mechanisms relevant to immunity. Recently, *P. fulvidraco* has been employed as an experimental model fish to investigate breeding techniques, development, lipid metabolism, immunology and toxicology (7–11). *Vibrio cholerae*, is an aquatic, gram-negative bacterium, which is responsible for pandemics of cholera disease worldwide (12). To decrease the danger of aquaculture, understanding molecular defense mechanisms of yellow catfish against microbial diseases is indispensable to establish reasonable control measures.

High-throughput RNA-sequencing (RNA-Seq) is a low-cost technique with high sensitivity, wide genome coverage, and unbiased quantification of transcript expression which is a relatively new technique that allows transcriptome analysis (13). Transcriptome analysis is widely used in genome research and functional gene identification, particularly for understanding hereditary responses to pathogens (14). In addition, transcriptome research has been increasingly applied to many fields of research including physiology, ecology, evolution and genetics, and has been critical for gene expression analysis, genetic marker mining, and gene discovery in many species (15). Recently, a variety of comparative transcriptomic studies focusing on fish immune responses after exposure to microbial pathogens were performed, such as in crucian carp (16), rainbow trout (17), half-smooth tongue sole (18), blunt snout bream (19), tilapia (20), soiny mullet (21), and miiuy croaker (22).

In this study, based on the head kidney of *P. fulvidraco* from PBS-control and *V. cholerae* infected groups, we established two transcriptome sequence libraries. Similar to the mammalian adrenal gland, the head kidney is an important endocrine and haematopoietic-lymphoid organ which is unique for teleost fish and participates in innate immune responses to pathogen infections (23). By comparison among the libraries, differentially expressed genes (DEGs) were recognized. Through gene ontology (GO) enrichment and Kyoto Encyclopedia of Genes and Genomes (KEGG) enrichment, these DEGs were enriched and the immune-related DEGs were identified that maybe provide insights into the potential molecular immune mechanisms of *P. fulvidraco*.

## Materials and methods

### Experimental fish models

Yellow catfish were collected from the aquatic market, Yancheng, Jiangsu Province, China, in October 2020 and were acclimatized in tanks at 24°C for two weeks before the experiment commenced. The fish were then divided into two groups, each containing three fish: a control group and the *V. cholerae* group. The control group was injected with 100 µL PBS whilst the *V. cholerae* group were injected with the same dose of bacterial suspension that had a final concentration of 1 × 10^7^ CFU/mL and were treated as the experimental group. The head kidneys were gathered 6 h after injection and stored at -80°C.

### RNA extraction, library construction and Illumina sequencing

Library constructions were performed at the Novogene Experimental Department (Beijing, China). RNA -easy isolation reagent (Vazyme, China) was used to extract the total RNA content of the head kidney. RNA degradation, purity, and contamination were measured by 1% agarose gels, NanoPhotometer^®^ spectrophotometer (IMPLEN, CA, USA), and Qubit^®^ 2.0 Fluorometer. RNA integrity was evaluated by the Agilent Bioanalyzer 2100 system (Agilent Technologies, CA, USA). We made use of the NEBNext^®^ Ultra™ RNA Library Prep Kit for Illumina^®^ (NEB, USA) to generate sequencing libraries. Random hexamers primers and M-MuLV were utilized for first strand cDNA synthesis, using DNA Polymerase I and RNase H for synthesis of the second strand. Purification of the PCR products involved the following steps: purification, adapter addition, and cDNA fragment selection, and PCR by universal PCR primers and Index (X) Primers. Finally, the insert size of the library from the libraries was sequenced using an Illumina HiSeq platform at Novogene.

### Sequencing data processing and assembly

Through in-house Perl scripts, we first processed raw reads of FASTA formatted sequences. In this procedure, by deleting reads such as those generated by adapters, clean reads were obtained, consisting of poly-N and low-quality reads from raw reads. Then, to obtain the reference sequence for subsequent analysis, we concatenated the clean reads. By using Trinity software, the compilation of *de novo* transcriptome data was executed by three independent components: Butterfly, Chrysalis, and Inchworm (24). Finally, the longissimus transcript of every gene was named unigenes. Meanwhile, based on clean reads with high quality, Q-value 20, Q-value 30, GC-content, and sequence duplication level of the clean reads were calculated.

### Unigenes annotation and functional enrichment

Unigenes were annotated by comparison to seven databases and processed by BLAST. The databases included: NT (NCBI non-redundant nucleotide sequences, e-value=1e-5), NR (NCBI non-redundant protein database, e-value=1e-5), Eukaryotic Orthologous Groups (KOG/COG) (http://www.ncbi.nlm.nih.gov/COG/, e-value=1e-3), Pfam (http://pfam.sanger.ac.uk/, e-value=0.01), GO (http://www.geneontology.org/, e-value=1e-6) (25), KEGG (http://www.genome.jp/kegg, e-value=1e-10), and Swiss-prot (http://www.ebi.ac.uk/uniprot/, e-value=1e-5). GO annotation of unigenes was derived from NR annotation by the Blast2GO program, and WEGO software was used to perform GO functional classification (26). Pathway assignments were executed using the KEGG database (27).

### Identification of DEGs

To estimate DEGs between two datasets, expression levels of unigenes were calculated using the FPKM method, the universal method for detecting gene expression (28). For the sake of calculating the expression profiles between PBS and *V. cholerae-*injected head kidney tissues, we made use of RSEM software to map the clean data (29). We obtained a read count for each gene from the mapping results. Before DEGs analysis, all read counts were normalized using edgeR (30). DEGs analysis of two libraries was carried out using the DEGseq R package (31). The *p*-value was adjusted according to the q-value. *Q*-value<0.005 and |log_2_(fold change)|>1 were assigned as differential expression genes. Then DEGs were subjected to KEGG pathway analysis and GO functional enrichment analysis. By Wallenius noncentral hypergeometric distribution, we employed GOseqR to implement GO enrichment analysis of DEGs, which can be adjusted for gene length bias in DEGs (32). KOBAS software was used to test the statistical enrichment of DEGs in the KEGG pathway (33).

### qRT-PCR of candidate gene expression

qRT-PCR was employed to check the expression level of the 10 candidate DEGs. Following the manufacturer’s instructions, MightyScript Plus First Strand cDNA Synthesis Master Mix (gDNA digester) (Sangon, China) was used to produce synthesized cDNA that acted as a template for qRT-PCR. Primer premier 5.0 software was then used to design candidate DEGs primers with reference to the cDNA sequence. The β-actin gene from *P. fulvidraco* was used as an internal reference. The sequences of PCR primers are shown in **Table 1**. By using Mastercycler ep realplex (Eppendorf, Germany) with the AceQ Universal SYBR qPCR Master Mix (Vazyme, China), qRT-PCR was performed in 20 µl reaction mixtures consisting of 10 µl 2×SYBR Green mix, 1 µl cDNA, 7 µl RNase-free H_2_O, and 1 µl forward and reverse primers. The steps of PCR were as follows: 20 s at 95°C, followed by 40 cycles of 15 s at 95°C and 15 s at 55°C, then at 72°C 20 s and 60°C for 1 min. Each independent experiment was performed in triplicate and determined the relative expression levels of the genes which was calculated with the 2^-△△CT^ comparative threshold cycle (Ct) method (34).

**Table 1:** Primers used for qRT-PCR in this paper.

Primer name	Forward Primer (5’-3’)	Reverse Primer (5’-3’)	Purpose
cadherin-5	GTGAACGCAACCAACGTCTT	CCCACAGGCTCATCCTCATT	qRT-PCR
CC chemokine SCYA101	GCTGCAAGAAGACGAGTTGA	CCATCGCTCTAGCCAGTAAG	qRT-PCR
calumenin	AGAGTGGGTGAAGACAGAGCG	TTCGTACAGCAGGTGTTTGG	qRT-PCR
C-type lectin	TATTCTGGTCGCTGCTACTTG	AGCCCTTATCTGCTGATATTCA	qRT-PCR
interleukin-13 receptor	TGGGTCTTCTGAGGTGGGATA	GTTGCTTGTTGCCAGTGAGG	qRT-PCR
Integrin	GAACTCGCTCAGGGAGGATT	ATGCGTTGATGAATGAAGTGTTT	qRT-PCR
LAMP 1	CAGCACTTTCACCCTCAACA	AACAACCCTGGAAACAAACAT	qRT-PCR
Mucin-2	CACCATATTCAGACCGTCAT	CAGTTCATCGTACAGCACCT	qRT-PCR
Natterin	TGTAAGACTCAGCACCACCC	GAGAATCACCTTCCCAAACC	qRT-PCR
Nramp	TCGCCTTCAACCTGCTGTCT	CAATCCCAACTGCCTGCTCT	qRT-PCR
β-actin	GCACAGTAAAGGCGTTGTGA	ACATCTGCTGGAAGGTGGAC	qRT-PCR

### Statistical analysis

Statistical analysis was used to examine the differences of mRNA expression levels and performed using the statistical software SPSS 17.0 and *p*<0.05 results were considered significant. All quantitative data were expressed as means ± SD at least three independent experiments.

## Results and discussion

### Sequencing and *de novo* assembly

To acquire the transcriptomic analysis data of the *P. fulvidraco* head kidney following injection of *V. cholerae*, we employed Illumina HiSeq sequencing platform to construct two libraries, and the results are summarized in **Table 2** (13). Approximately, 53,492,522 and 47,733,280 clean reads were noted from the yellow catfish head kidney in *V. cholerae* challenged and PBS-challenged fish. Each sample had a quality score over 96% and 91% with Q-value 20 and Q-value 30, respectively, and that the GC content of each library was approximately equal (44%). We spliced all the clean data into 135,097 transcripts (total length of 226,790,028) with a mean length of 1,697, N50 length of 2,854, and N90 length of 677 (**Table 3**). We further assembled 45,544 unigenes (total length of 62,537,256) with an average length of 1,373, N50 length of 1,439, and N90 length of 536. These results show that the transcriptome data were reliable, and the sequence data were high-quality.

**Table 2:** The quality of raw and clean reads.

Sample	Raw Reads	Clean Reads	Clean Bases	Error(%)	Q20(%)	Q30(%)	GC Content(%)
PBS	54,703,956	53,492,522	8.02G	0.03	96.93	92.43	44.74
*V. cholerae*	48,686,580	47,733,280	7.16G	0.03	96.7	91.63	45.6

**Table 3:** Distribution of splicing length.

	Total nucleotide	Min length	Mean length	Median length	Max length	N50	N90
Transcripts	226,790,028	301	1679	1024	19,263	2854	677
unigenes	62,537,256	301	1373	727	19,263	1439	536

**Figure 1:**
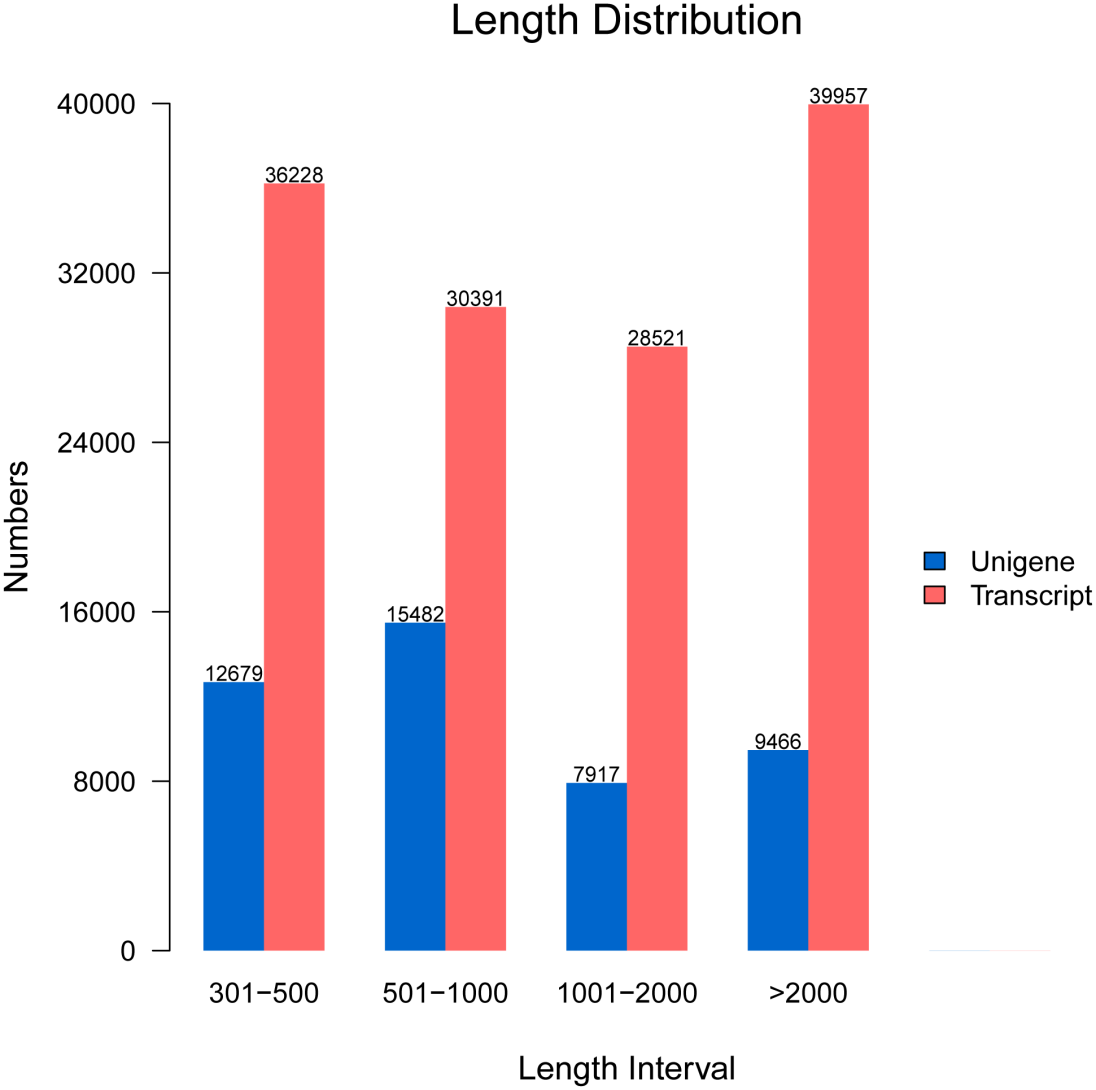
The line plot shows the sequence length distribution of the unigenes and transcripts.

After assembly, the length of unigenes extended from 301 bp to 19,263 bp with a length of 1,373 bp on average. The length of assembled unigenes indicated that 12,679 of unigenes extended from 300 bp to 500 bp, 15,482 of unigenes extended from 500 bp to 1000 bp, 7,917 of unigenes extended from 1,000 bp to 2,000 bp, and 9,466 of unigenes were more than 2,000 bp. In addition, the length of transcripts showed that 30,391 transcripts ranged from 500 bp to 1,000 bp, 36,228 of transcripts ranged from 300 bp to 500 bp, 28,521 of transcripts ranged from 1,000 bp to 2,000 bp, and 39,957 of transcripts exceeded 2,000 bp (**Figure 1**).

### Annotation of assembled unigenes

**Figure 2:**
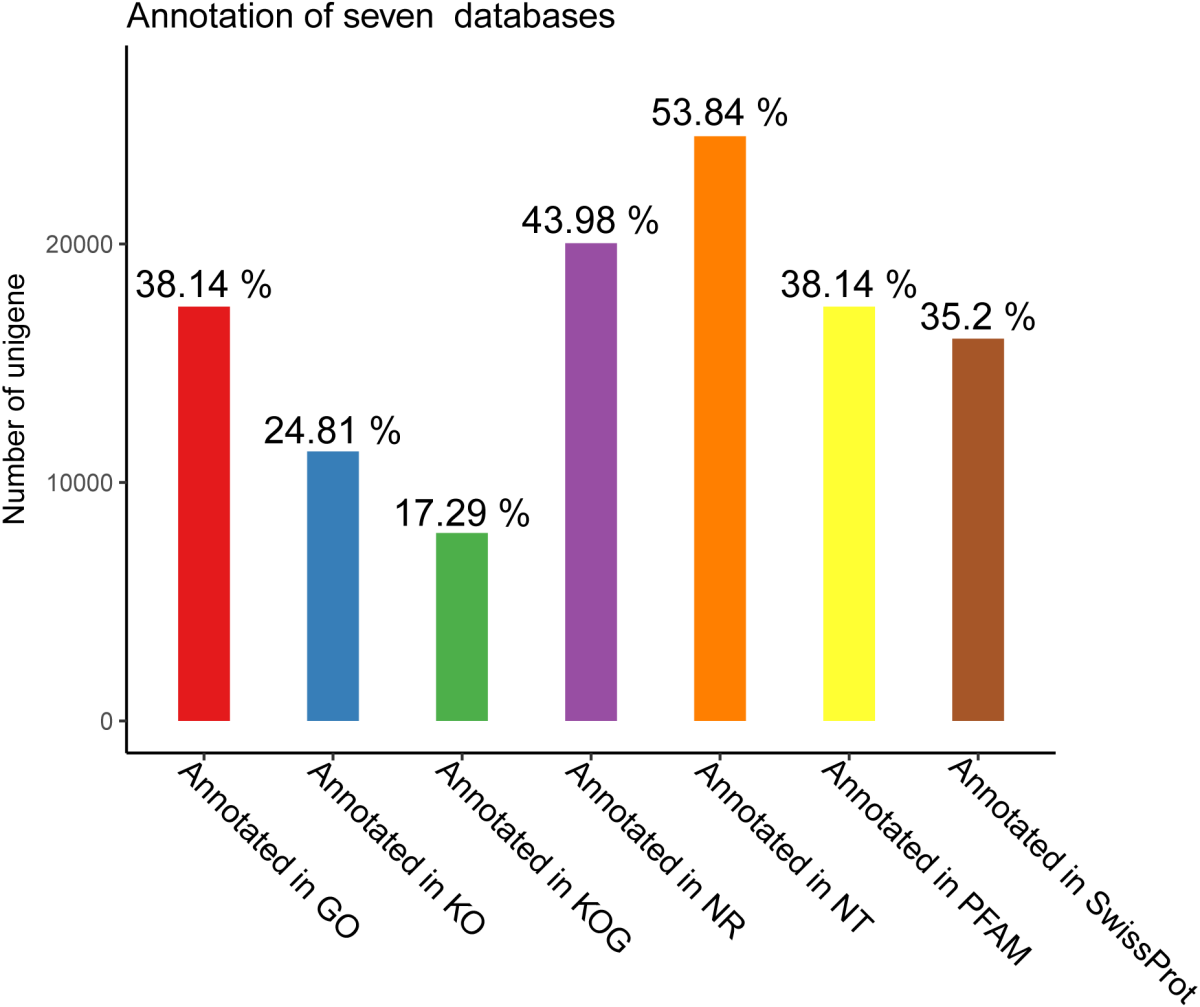
Annotated unigenes in seven databases.

**Figure 3:**
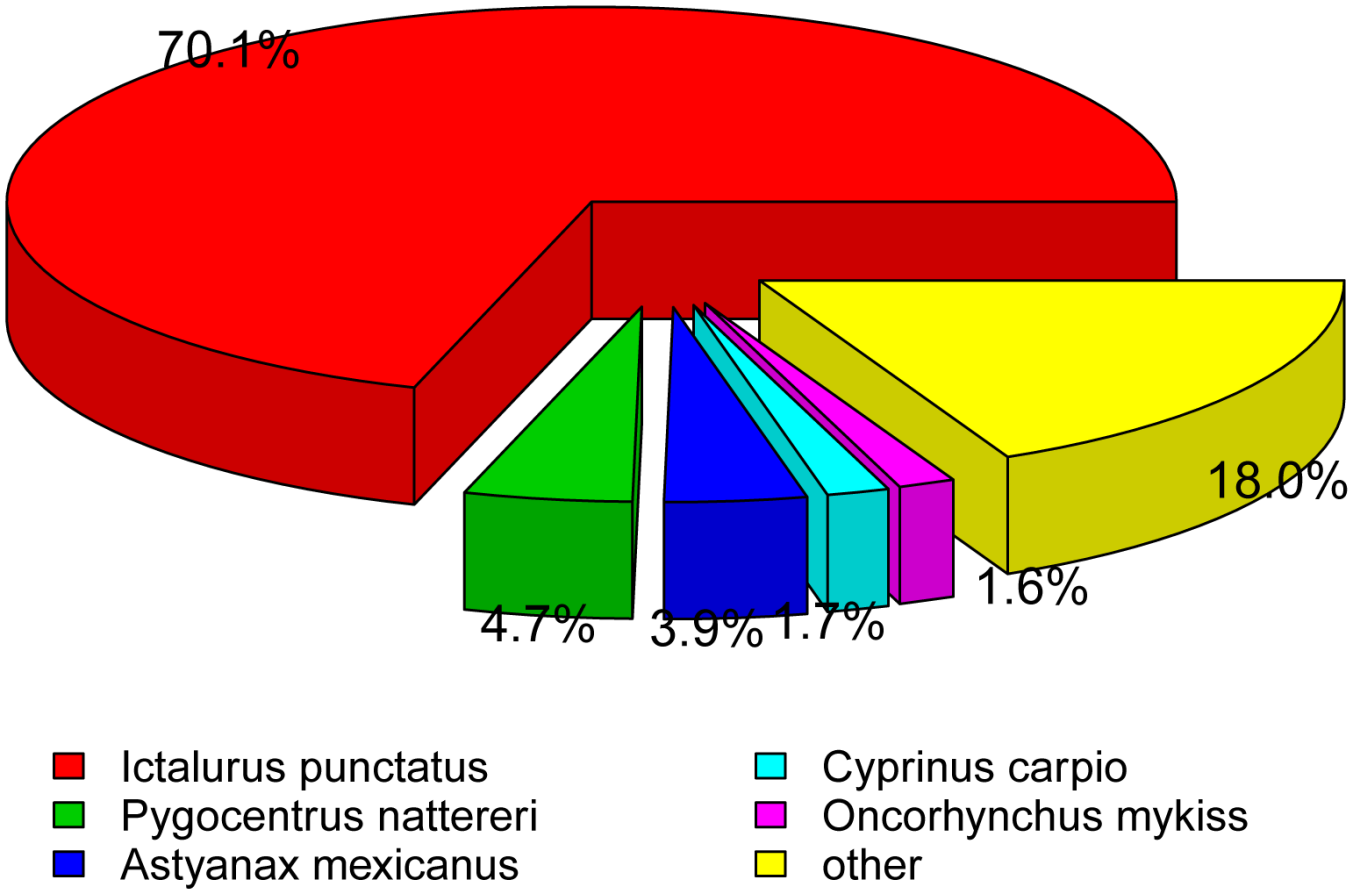
Map of species distribution in the NR database.

To gain a comprehensive view of gene function information, 45,544 unigenes were annotated by seven databases including NR, NT, Swiss-Port, KOG, PFAM, GO, and KO (35). Of these unigenes, 29,004 unigenes (63.68%) were annotated in one database at least, and 5,544 unigenes (12.17%) were constructed in all databases. The number of unigenes that may be annotated in diverse databases was 24,521 in NR (53.84%), 20,034 in NT (43.98%), 11,300 in KO (24.81%), 16,032 in Swiss-Prot (35.20%), 17,372 in PFAM (38.14%), 17,372 in GO (38.14%), and 7,878 in KOG (17.29%) (**Figures 2**, **3** and **Table 4**).

**Table 4:** Statistics of annotation.

	Number of genes	Percentage (%)
Annotated in NT	20034	43.98
Annotated in NR	24521	53.84
Annotated in KO	11300	24.81
Annotated in SwissProt	16032	35.2
Annotated in PFAM	17372	38.14
Annotated in GO	17372	38.14
Annotated in KOG	7878	17.29
Annotated in all Databases	5544	12.17
Annotated in at least one Database	29004	63.68
Total Unigenes	45544	100

**Figure 4:**
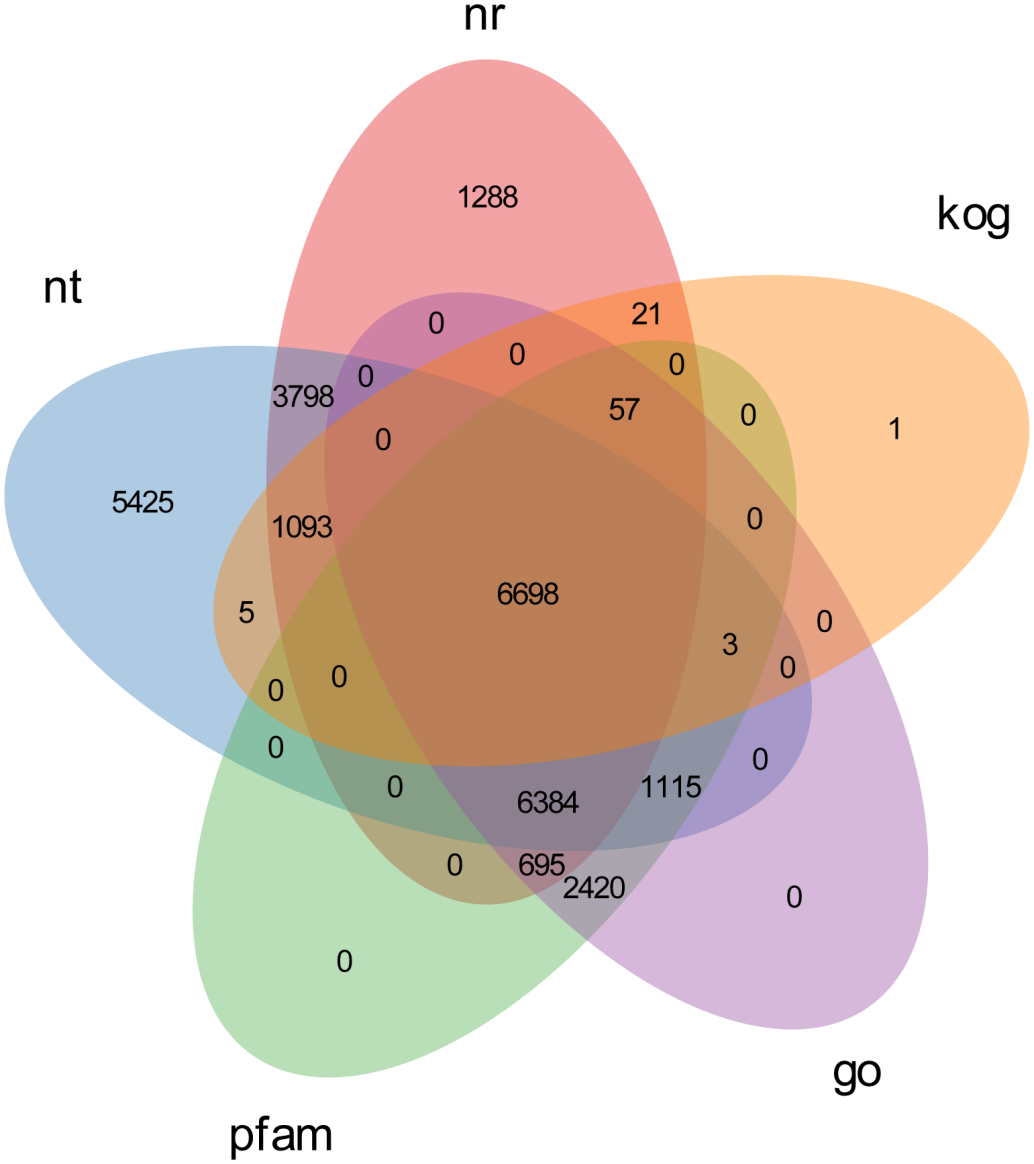
Venn diagram of overlap of the seven databases.

Using a BLAST search compared to the NR database, matching these unigenes by species classification revealed that the number of hits between *P. fulvidraco* and *Ictalurus punctatus* were the highest (70.1%), followed by *Pygocentrus nattereri* (4.7%), *Astyanax mexicanus* (3.9%), *Cyprinus carpio* (1.7%), *Oncorhynchus mykiss* (1.6%), and other vertebrates (18.0%) (**Figure 4**).

### Functional annotation of genes

**Figure 5:**
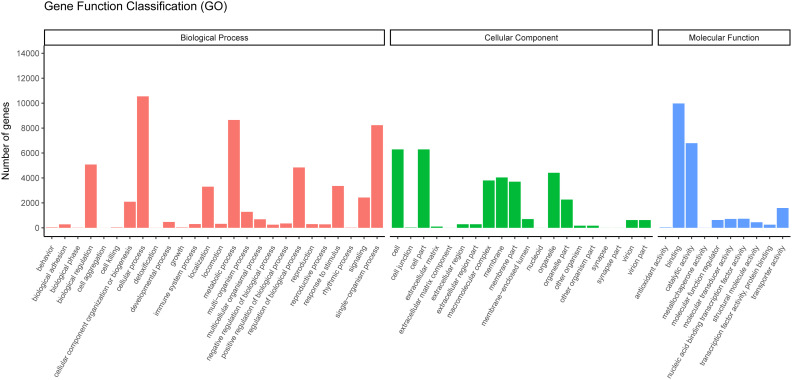
Gene ontology classifications of assembled unigenes. A total of 17,372 unigenes were categorized into three functional categories: biological process, cellular component, and molecular function.

GO is widely used to classify genes and gene products over species (36). To further study the biological importance of the unigenes, we made use of Blast2GO to determine the functional categories (25). According to the NR annotation from NCBI, 17,372 unigenes were classified into three major categories (cellular components, biological processes, and molecular functions) and 55 subcategories. In the biological process category, most unigenes were assigned to single-organism processes, metabolic process subcategories and cellular processes. Many unigenes were associated with the cell fraction and cell, in the cellular component category. In the molecular function category, most of the unigenes were found to participate in binding and catalytic activity (**Figure 5**).

**Figure 6:**
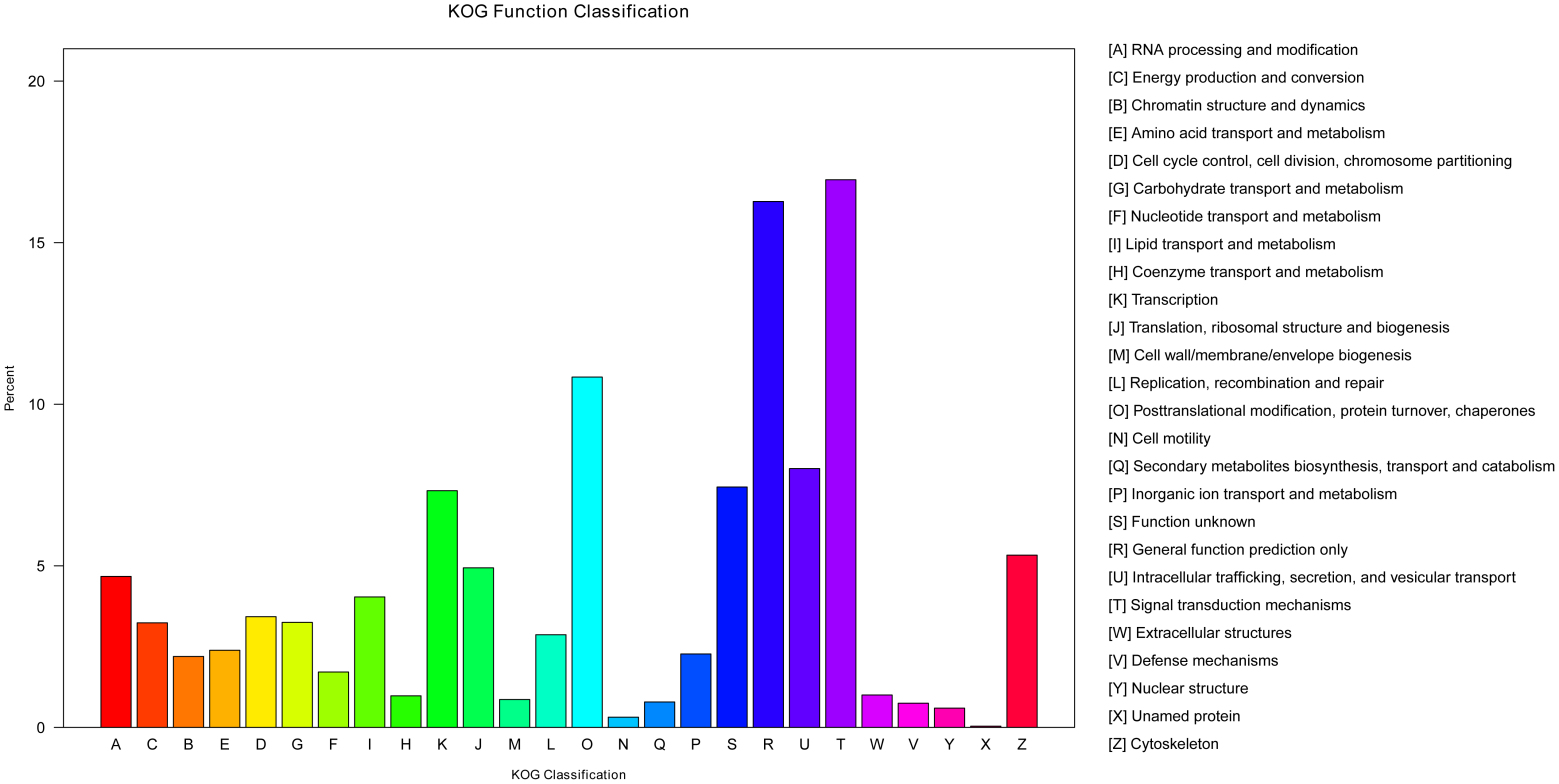
Function annotations of unigenes by KOG.

The KOG is a database that can classify isogenous gene products. It is assumed that each KOG protein comes from an ancestor protein (37). To predict their possible functions and classify them by the KOG database, 7,878 unigenes were classified into 26 functional categories (**Figure 6**). Among these, the signal transduction mechanisms (T, 1,335) included the most unigenes, followed by general function prediction only (R, 1,282) and post-translational modification, protein turnover, and chaperones (O, 854). A number of unigenes (V, 59) were classified to the defense mechanisms category, which indicated these unigenes may participate in immunity protection of yellow catfish. The unigenes resembled those acquired from the *Megalobrama amblycephala* (38).

The KEGG is a bioinformatic database for the systematic analysis of gene functions (39). In this study, 11,300 unigenes in total were designated to 232 known pathways. The largest group was “PI3K-Akt signaling pathway”, which contains 351 unigenes, followed by “Endocytosis” (330), “MAPK signaling pathway” (286), “Focal adhesion” (271), and “Rap1 signaling pathway” (257) (**Table 5**). Moreover, these 232 pathways were divided into five categories: environmental information processing, cellular processes, metabolism and organismal systems, and genetic information processing (**Figure 7**). Within the organismal systems, “endocrine system” (771) and “immune system” (785) were the two richest subcategories. In the metabolism category, the most common categories were “amino acid metabolism” (252), “lipid metabolism” (331) and “carbohydrate metabolism” (320). In the signal transduction category, the unigenes were mostly associated with environmental Information Processing (1686).

**Table 5:** KEGG annotation of assembled unigenes.

#	Pathway	Pathway ID	Number of unignes
1	PI3K-Akt signaling pathway	ko04151	351
2	Endocytosis	ko04144	330
3	MAPK signaling pathway	ko04010	286
4	Focal adhesion	ko04510	271
5	Rap1 signaling pathway	ko04015	257
6	Regulation of actin cytoskeleton	ko04810	251
7	Ras signaling pathway	ko04014	225
8	cAMP signaling pathway	ko04024	225
9	cGMP - PKG signaling pathway	ko04022	193
10	Chemokine signaling pathway	ko04062	187
11	Tight junction	ko04530	186
12	Axon guidance	ko04360	185
13	Oxytocin signaling pathway	ko04921	185
14	Calcium signaling pathway	ko04020	181
15	Thyroid hormone signaling pathway	ko04919	175
16	Protein processing in endoplasmic reticulum	ko04141	174
17	Purine metabolism	ko00230	174
18	Insulin signaling pathway	ko04910	173
19	Phagosome	ko04145	172
20	Hippo signaling pathway	ko04390	170
21	FoxO signaling pathway	ko04068	163
22	Platelet activation	ko04611	162
23	RNA transport	ko03013	160
24	Cytokine-cytokine receptor interaction	ko04060	154
25	Adrenergic signaling in cardiomyocytes	ko04261	153
26	Vascular smooth muscle contraction	ko04270	152
27	Ubiquitin mediated proteolysis	ko04120	150
28	Lysosome	ko04142	148
29	Neurotrophin signaling pathway	ko04722	147
30	AMPK signaling pathway	ko04152	146
31	Fc gamma R-mediated phagocytosis	ko04666	145
32	Signaling pathways regulating pluripotency of stem cells	ko04550	144
33	Wnt signaling pathway	ko04310	144
34	Sphingolipid signaling pathway	ko04071	141
35	Cell cycle	ko04110	140
36	Dopaminergic synapse	ko04728	139
37	Oocyte meiosis	ko04114	137
38	Spliceosome	ko03040	134
39	Osteoclast differentiation	ko04380	134
40	Oxidative phosphorylation	ko00190	132
41	Glucagon signaling pathway	ko04922	131
42	HIF-1 signaling pathway	ko04066	128
43	Leukocyte transendothelial migration	ko04670	126
44	Phosphatidylinositol signaling system	ko04070	124
45	Adherens junction	ko04520	123
46	Ribosome	ko03010	122
47	GnRH signaling pathway	ko04912	122
48	Carbon metabolism	ko01200	121
49	T cell receptor signaling pathway	ko04660	121
50	Neuroactive ligand-receptor interaction	ko04080	119
51	Estrogen signaling pathway	ko04915	118
52	Natural killer cell mediated cytotoxicity	ko04650	116
53	Jak-STAT signaling pathway	ko04630	115
54	Melanogenesis	ko04916	115
55	Cholinergic synapse	ko04725	113
56	TNF signaling pathway	ko04668	111
57	Circadian entrainment	ko04713	110
58	Glutamatergic synapse	ko04724	110
59	NF-kappa B signaling pathway	ko04064	107
60	Cell adhesion molecules (CAMs)	ko04514	107
61	Inflammatory mediator regulation of TRP channels	ko04750	104
62	B cell receptor signaling pathway	ko04662	103
63	ErbB signaling pathway	ko04012	101

**Figure 7:**
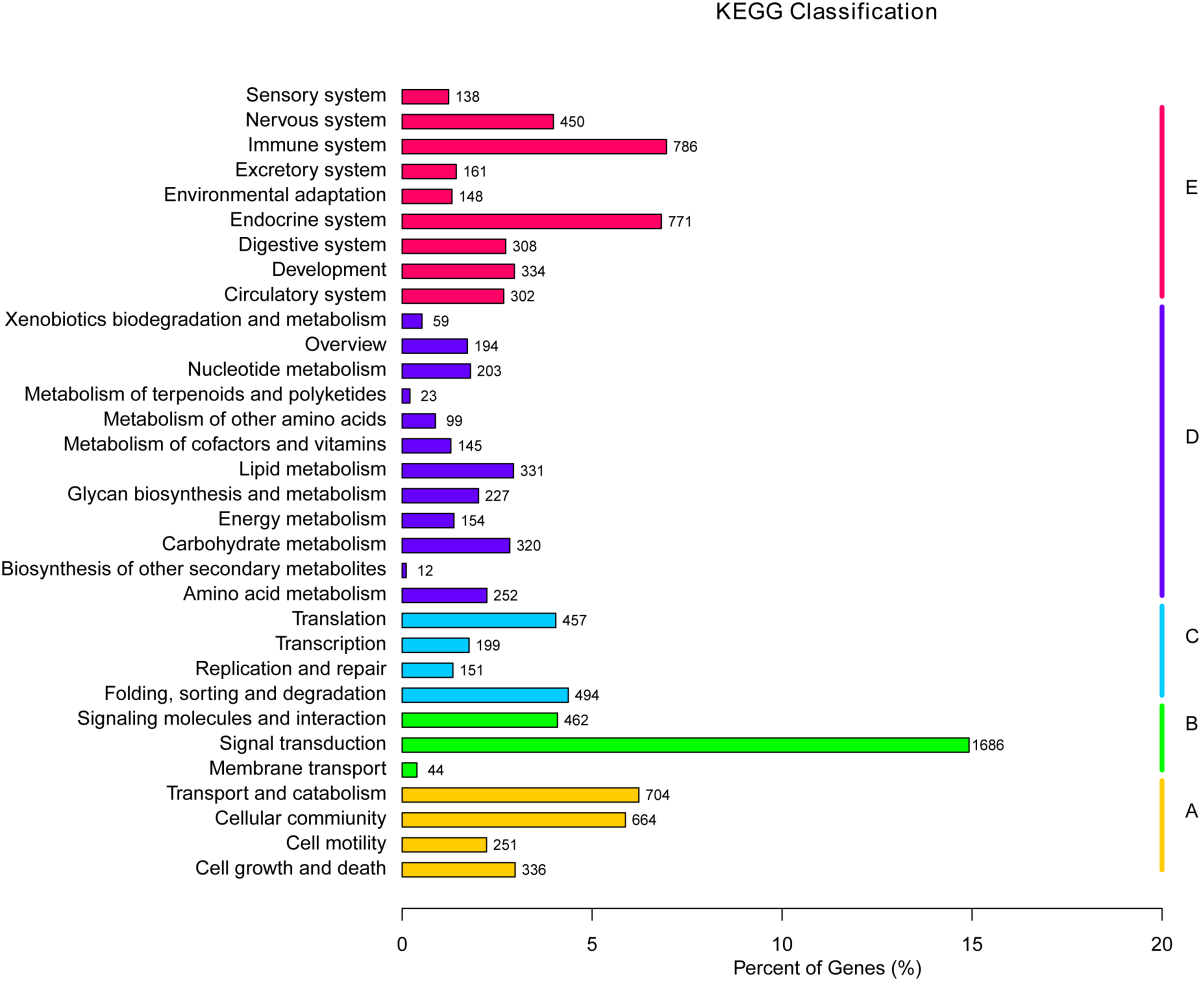
KEGG pathway assignment of the assembled unigenes. Different colored bars represent different groups.

### Identification of DEGs analysis

**Figure 8:**
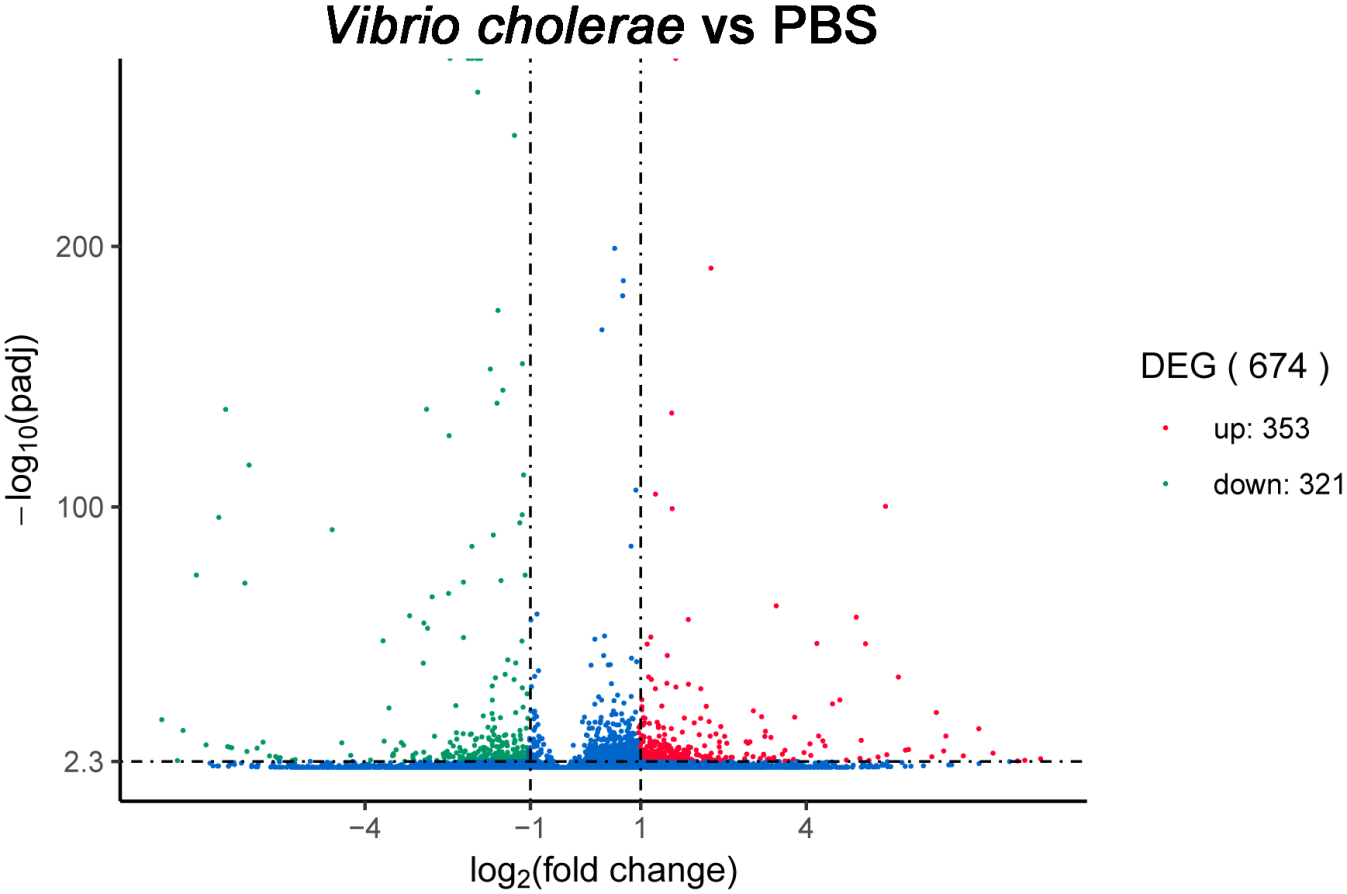
Volcano plot of the differentially expressed genes in head kidney of yellow catfish.

To recover the expression levels in the PBS- and *V. cholerae*-injected libraries, all unigenes were analyzed by RSEM software, and DEG-seq was applied to determine the DEGs when the Q-value < 0.005 and |log_2_(fold change)|>1. A total of 674 differential expression genes were induced, of which 352 were significantly upregulated and 321 were significantly downregulated unigenes (**Figure 8**).

### DEGs analysis

**Figure 9:**
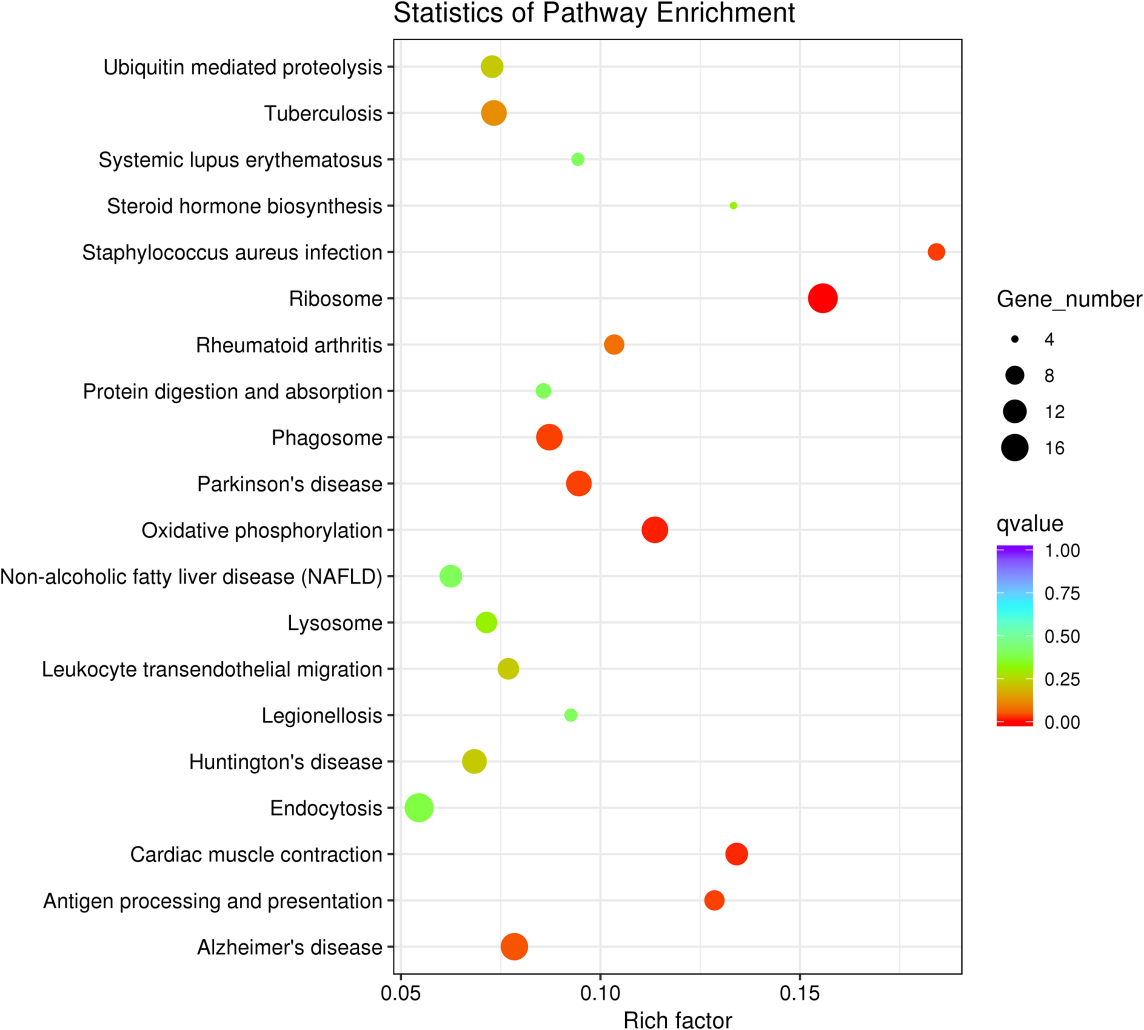
Top 20 significantly enriched KEGG pathways of DEGs.

To further investigate the function of DEGs implicated in the immune response of *P. fulvidraco* challenged with *V. cholerae*, DEGs were compared with the KEGG database for pathway enrichment to understand the pathway, and 210 pathways in total were annotated. The top 20 of these divided pathways were shown in **Table 6** and **Figure 9**. The largest quantities of DEGs appeared in the category “Ribosome”, followed by “Endocytosis”, and “Oxidative phosphorylation”. Additionally, from 11 immune-related signal pathways, we identified 29 genes, including “Antigen processing and presentation” (9 DEGs), “Fc gamma R-mediated phagocytosis” (2 DEGs), “Leukocyte transendothelial migration” (10 DEGs), “TNF signaling pathway” (3 DEGs), “Toll-like receptor signaling pathway” (1 DEG), “Chemokine signaling pathway” (2 DEGs), “MAPK signaling pathway” (1 DEG), “NOD-like receptor signaling pathway” (2 DEGs), “Platelet activation” (2 DEGs), “Intestinal immune network for IgA production” (3 DEGs), and “NF-kappa B signaling pathway” (3 DEGs).

**Table 6:** KEGG annotation of assembled unigenes.

pathway	ID	DEGs number
Antigen processing and presentation	ko04612	9
Fc gamma R-mediated phagocytosis	ko04666	2
Leukocyte transendothelial migration	ko04670	10
TNF signaling pathway	ko04668	3
Toll-like receptor signaling pathway	ko04620	1
Chemokine signaling pathway	ko04062	2
MAPK signaling pathway	ko04010	9
NOD-like receptor signaling pathway	ko04621	2
Lysosome	ko04142	10
Intestinal immune network for IgA production	ko04672	3
NF-kappa B signaling pathway	ko04064	3

### The validation of DEGs by qRT-PCR

**Figure 10:**
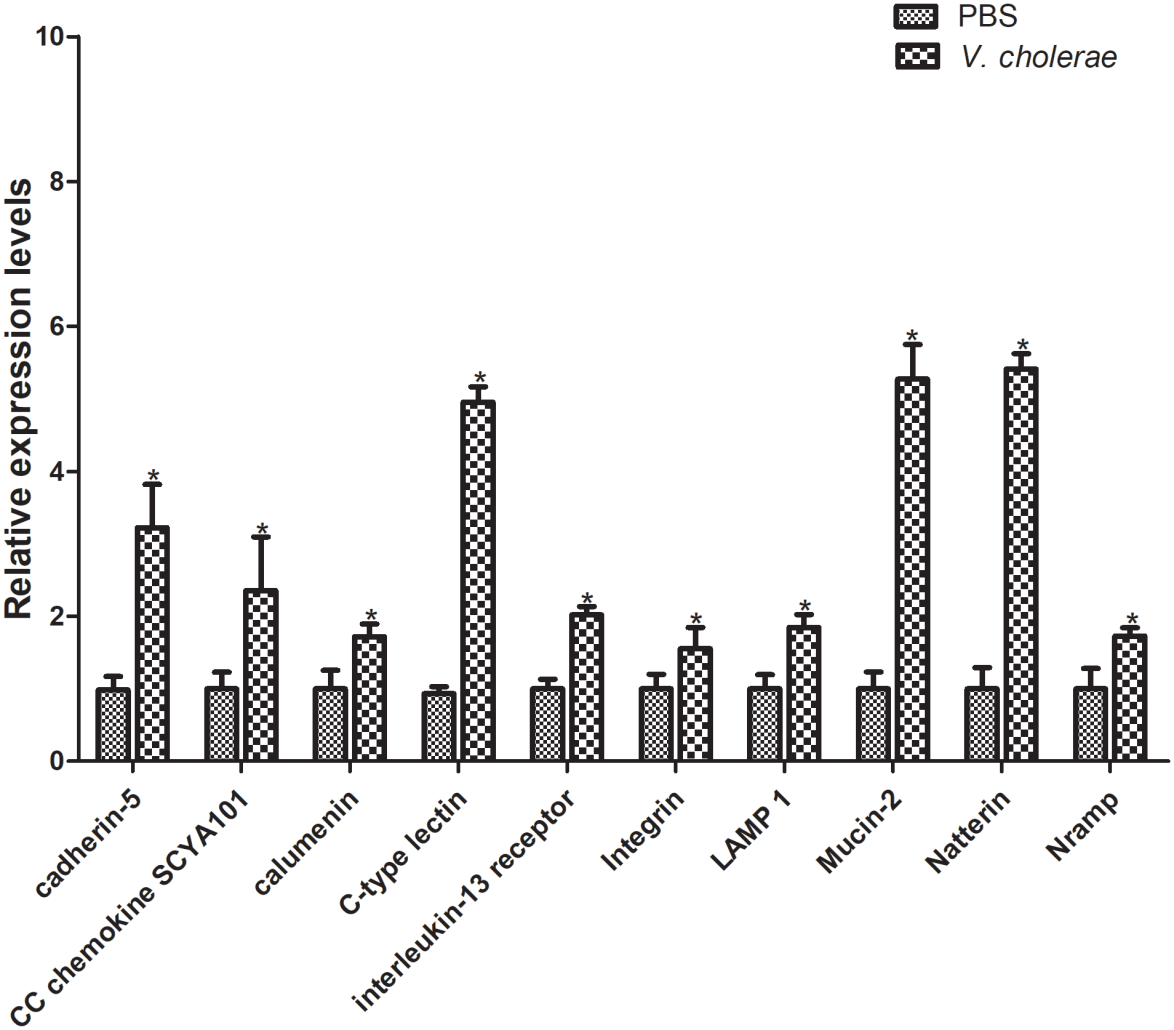
Validation of DEGs by qRT-PCR. The relative gene expression levels of 10 selected DEGs were examined. The β-actin gene was used as an internal standard. The means + SEM of three independent samples are shown (means ± SE, n = 3). Differences between treated and control samples were tested by one-way analysis of variance (ANOVA) followed by the Duncan’s new multiple range test. The *P* values are shown as *P < 0.05.

Furthermore, to verify the differentially expressed genes, 10 genes were selected for qPT-PCR analysis challenged by *V. cholerae* for 6 h. The results showed that, compared with the control group, most immune related genes were upregulated at different points (**Figure 10**). The cadherin gene family plays a key role in target recognition and cell adhesion (40). Compared with the control group, cadherin-5 expression was significantly increased by more than double in the experimental group. In addition, cadherin is a transmembrane cell adhesion molecule conserved in metazoan organisms and plays an important role in histomorphogenesis and homeostasis (41). CC chemokines are a group of 28 chemokines with N-terminal CC domain, which play key roles in regulating leukocyte transport to injury, infection, or inflammation sites (42). The CC chemokine SCYA101 gene in *P. fulvidraco* was overexpressed by more than two-fold in response to *V. cholerae*. Moreover, calumenin (calu) is a well-conserved multi-EF-hand-containing Ca^2+^-binding protein, which may provide a new target for bladder cancer and has multiple mechanisms, including TME remodeling, gene mutation, and ferroptosis (43). After injection of *V. cholerae*, the expression level of calu was higher than that of the control group. Additionally, C-type lectins (CTLs) are calcium-dependent, carbohydrate-binding, pattern recognition receptors that play important roles in the immune system and primarily bind carbohydrate-based or other ligands that are involved in recognizing pathogens and cell adhesion (44–46). Compared with PBS injection, expression of CTLs was increased by over 5-fold. As a closely related class I cytokine, Interleukin (IL)- 13 plays important roles in the T helper (Th)-2 immune response *via* heterodimeric receptors (47). The integrin family is included in an assortment of cell processes, including cell proliferation, separation, and migration, and in the pathogenesis of disease (48). Lysosomal membrane glycoprotein 1 (LAMP 1) is an integral membrane protein of the lysosome, which plays an important role in lysosomal biogenesis, autophagy, immune response, and cholesterol homeostasis (49). Mucin is synthesized and secreted by epithelial goblet cells, and is a key component of the innate immune system and plays an important part within the digestive system (50). The natterin protein family was first found in the venom of thalassophryne nattereri, a fish with medical significance. In the past decade, natterin-like genes have been found in various organisms, including genes that perform immune related functions (51). In conclusion, these immune genes may be related to the immune defense of *P. fulvidraco* after bacterial infection.

## Conclusion

*P. fulvidraco* is a famous fresh-water fish with enormous economic value in eastern China. Raising a large number of *P. fulvidraco* leads to the outbreak of various diseases and thus, economic losses. In this study, the transcriptomic characteristics following *V. cholerae* injection in the liver of *P. fulvidraco* were analyzed in detail. We identified a great deal of genes and pathways involved in the immunization to *V. cholerae* in *P. fulvidraco.* A total of 674 DEGs were identified, including 321 downregulated genes and 352 upregulated genes, some of which are related to “Antigen processing and presentation”, “Fc gamma R-mediated phagocytosis”, “Toll-like receptor signaling pathway” and other signaling pathways. These results were obtained for the first time in this work. In conclusion, this study may provide an opportunity to explore the immune defense mechanism of *P. fulvidraco* against bacterial attack.

## Data availability statement

The datasets presented in this study can be found in online repositories. The names of the repository/repositories and accession number(s) can be found below: https://www.ncbi.nlm.nih.gov/genbank/, PRJNA902142.

## Author contributions

The authors’ responsibilities were as follows: S-HJ and Q-NL: Analyzed data, Wrote the manuscript. L-XW, Y-TC, H-BZ, and R-TM: Analyzed data and conducted experiments. D-ZZ: Resources. Q-NL: Funding acquisition. B-PT and L-SD: Experiment design and manuscript review and editing, Resources. All authors contributed to the article and approved the submitted version.

## References

[1] Yang RB Xie CX Fan QX Gao C Fang LB. Ontogeny of the digestive tract in yellow catfish *Pelteobagrus fulvidraco* larvae. Aquaculture (2010) 302(1):112–23. doi: 10.1016/j.aquaculture.2010.02.020

[2] Gong G Dan C Xiao S Guo W Huang P Xiong Y. Chromosomal-level assembly of yellow catfish genome using third-generation DNA sequencing and Hi-c analysis. Gigascience (2018) 7(11):giy120. doi: 10.1093/gigascience/giy120 30256939 PMC6228179

[3] Hu YH Zhou X Jiang XX Zhang GR Shi ZC Ji W. Molecular characterization, expression analysis and function identification of Pf_TNF-α and its two receptors Pf_TNFR1 and Pf_TNFR2 in yellow catfish (Pelteobagrus fulvidraco). Int J Biol Macromol (2021) 185:176–93. doi: 10.1016/j.ijbiomac.2021.06.090 34144067

[4] Zhao H Wang G Wang H Mo W Huang Y Cao J. Effects of dietary sodium butyrate on growth, digestive enzymes, body composition and nutrient retention-related gene expression of juvenile yellow catfish (*Pelteobagrus fulvidraco*). Anim Nutr (2021) 7(2):539–47. doi: 10.1016/j.aninu.2020.12.007 PMC8245809 34258443

[5] Liu Y Wu PD Zhang DZ Zhang HB Tang BP Liu QN. Mitochondrial genome of the yellow catfish *Pelteobagrus fulvidraco* and insights into bagridae phylogenetics. Genomics (2019) 111(6):1258–65. doi: 10.1016/j.ygeno.2018.08.005 30118781

[6] Lu SF Zhao N Zhao A He RG. Effect of soybean phospholipid supplementation in formulated microdiets and live food on foregut and liver histological changes of *Pelteobagrus fulvidraco* larvae. Aquaculture (2008) 278:119–27. doi: 10.1016/j.aquaculture.2007.12.007

[7] Liu HQ Guan B Xu J Hou CC Tian H Chen HX. Genetic manipulation of sex ratio for the large-scale breeding of YY super-male and XY all-male yellow catfish (*Pelteobagrus fulvidraco* (Richardson)). Mar Biotechnol (2013) 15:321–8. doi: 10.1007/s10126-012-9487-7 23053056

[8] Zheng JL Luo Z Zhu QL Tan XY Chen QL Sun LD. Molecular cloning and expression pattern of 11 genes involved in lipid metabolism in yellow catfish pelteobagrus fulvidraco. Gene (2013) 531(1):53–63. doi: 10.1016/j.gene.2013.08.028 23988502

[9] Zhou X Zhang GR Ji W Shi ZC Ma XF Luo ZL. The dynamic immune response of yellow catfish (*Pelteobagrus fulvidraco*) infected with edwardsiella ictaluri presenting the inflammation process. Front Immunol (2021) 12:625928. doi: 10.3389/fimmu.2021.625928 33732247 PMC7959794

[10] Chen K Zhao F Ouyang G Shi Z Ma L Wang B. Molecular characterization and expression analysis of Tf_TLR4 and Tf_TRIL in yellow catfish *Tachysurus fulvidraco* responding to *Edwardsiella ictaluri* challenge. Int J Biol Macromol (2021) 167:746–55. doi: 10.1016/j.ijbiomac.2020.11.196 33278446

[11] Wang S Li X Zhang M Jiang H Wang R Qian Y. Ammonia stress disrupts intestinal microbial community and amino acid metabolism of juvenile yellow catfish (*Pelteobagrus fulvidraco*). Ecotoxicol Environ Saf (2021) 227:112932. doi: 10.1016/j.ecoenv.2021.112932 34700169

[12] Das B Pazhani GP Sarkar A Mukhopadhyay AK Nair GB Ramamurthy T. Molecular evolution and functional divergence of vibrio cholerae. Curr Opin Infect Dis (2016) 29(5):520–7. doi: 10.1097/QCO.0000000000000306 27537830

[13] Dillies MA Rau A Aubert J Hennequet-Antier C Jeanmougin M Servant N. A comprehensive evaluation of normalization methods for illumina high-throughput RNA sequencing data analysis. Brief Bioinform (2013) 14(6):671–83. doi: 10.1093/bib/bbs046 22988256

[14] Mortazavi A Williams BA McCue K Schaeffer L Wold B. Mapping and quantifying mammalian transcriptomes by RNA-seq. Nat Methods (2008) 5(7):621–8. doi: 10.1038/nmeth.1226 PMC13303166 18516045

[15] Jiang Z Zhou X Li R Michal JJ Zhang S Dodson MV. Whole transcriptome analysis with sequencing: methods, challenges and potential solutions. Cell Mol Life Sci (2015) 72(18):3425–39. doi: 10.1007/s00018-015-1934-y PMC6233721 26018601

[16] Zhou Y Zhu L Sun Y Zhang H Wang J Qin W. Localization of RNA pol II CTD (S5) and transcriptome analysis of testis in diploid and tetraploid hybrids of red crucian carp (♀) × common carp (♂). Front Genet (2021) 12:717871. doi: 10.3389/fgene.2021.717871 34567072 PMC8458772

[17] Roh H Kim N Lee Y Park J Kim BS Lee MK. Dual-organ transcriptomic analysis of rainbow trout infected with ichthyophthirius multifiliis through Co-expression and machine learning. Front Immunol (2021) 12:677730. doi: 10.3389/fimmu.2021.677730 34305907 PMC8296305

[18] Lin G Gao D Lu J Sun X. Transcriptome profiling reveals the sexual dimorphism of gene expression patterns during gonad differentiation in the half-smooth tongue sole (Cynoglossus semilaevis). Mar Biotechnol (NY) (2021) 23(1):18–30. doi: 10.1007/s10126-020-09996-x 32996005

[19] Wang Z Jia Y Huang X Zhu D Liu H Wang W. Transcriptome profiling towards understanding of the morphogenesis in the scale development of blunt snout bream (*Megalobrama amblycephala*). Genomics (2021) 113(3):983–91. doi: 10.1016/j.ygeno.2020.12.043 33640463

[20] Zheng Y Wu W Hu G Qiu L Chen J. Transcriptome analysis of juvenile tilapia (*Oreochromis niloticus*) blood, fed with different concentrations of resveratrol. Front Physiol (2020) 11:600730. doi: 10.3389/fphys.2020.600730 33362577 PMC7755862

[21] Qi Z Wu P Zhang Q Wei Y Wang Z Qiu M. Transcriptome analysis of soiny mullet (*Liza haematocheila*) spleen in response to streptococcus dysgalactiae. Fish Shellfish Immunol (2016) 49:194–204. doi: 10.1016/j.fsi.2015.12.029 26707943

[22] Chu Q Song W Cui J Xu T. Genome-guided transcriptome analysis of miiuy croaker provides insights into pattern recognition receptors and cytokines in response to vibrio anguillarum. Dev Comp Immunol (2017) 73:72–8. doi: 10.1016/j.dci.2017.03.009 28315364

[23] Geven EJW Klaren PHM. The teleost head kidney: Integrating thyroid and immune signalling. Dev Comp Immunol (2017) 66:73–83. doi: 10.1016/j.dci.2016.06.025 27387152

[24] Grabherr MG Haas BJ Yassour M Levin JZ Thompson DA Amit I. Full-length transcriptome assembly from RNA-seq data without a reference genome. Nat Biotechnol (2011) 29(7):644–52. doi: 10.1038/nbt.1883 PMC3571712 21572440

[25] Conesa A Götz S García-Gómez JM Terol J Talón M Robles M. Blast2GO: A universal tool for annotation, visualization and analysis in functional genomics research. Bioinformatics (2005) 21(18):3674–6. doi: 10.1093/bioinformatics/bti610 16081474

[26] Ye J Zhang Y Cui H Liu J Wu Y Cheng Y. WEGO 2.0: a web tool for analyzing and plotting GO annotations, 2018 update. Nucleic Acids Res (2018) 46(W1):W71–5. doi: 10.1093/nar/gky400 PMC6030983 29788377

[27] Kanehisa M Furumichi M Tanabe M Sato Y Morishima K. KEGG: new perspectives on genomes, pathways, diseases and drugs. Nucleic Acids Res (2017) 45(D1):D353–61. doi: 10.1093/nar/gkw1092 PMC5210567 27899662

[28] Trapnell C Williams BA Pertea G Mortazavi A Kwan G van Baren MJ. Transcript assembly and quantification by RNA-seq reveals unannotated transcripts and isoform switching during cell differentiation. Nat Biotechnol (2010) 28(5):511–5. doi: 10.1038/nbt.1621 PMC3146043 20436464

[29] Li B Dewey CN. RSEM: accurate transcript quantification from RNA-seq data with or without a reference genome. BMC Bioinf (2011) 12:323. doi: 10.1186/1471-2105-12-323 PMC3163565 21816040

[30] Robinson MD McCarthy DJ Smyth GK. edgeR: a bioconductor package for differential expression analysis of digital gene expression data. Bioinformatics (2010) 26(1):139–40. doi: 10.1093/bioinformatics/btp616 PMC2796818 19910308

[31] Wang L Feng Z Wang X Wang X Zhang X. DEGseq: an r package for identifying differentially expressed genes from RNA-seq data. Bioinformatics (2010) 26(1):136–8. doi: 10.1093/bioinformatics/btp612 19855105

[32] Young MD Wakefield MJ Smyth GK Oshlack A. Gene ontology analysis for RNA-seq: accounting for selection bias. Genome Biol (2010) 11(2):R14. doi: 10.1186/gb-2010-11-2-r14 20132535 PMC2872874

[33] Mao X Cai T Olyarchuk JG Wei L. Automated genome annotation and pathway identification using the KEGG orthology (KO) as a controlled vocabulary. Bioinformatics (2005) 21(19):3787–93. doi: 10.1093/bioinformatics/bti430 15817693

[34] Livak KJ Schmittgen TD. Analysis of relative gene expression data using real-time quantitative PCR and the 2(-delta delta C(T)) method. Methods (2001) 25(4):402–8. doi: 10.1006/meth.2001.1262 11846609

[35] Chalifa-Caspi V. RNA-Seq in nonmodel organisms. Methods Mol Biol (2021) 2243:143–67. doi: 10.1007/978-1-0716-1103-6_8 33606257

[36] Gabaldón T Koonin EV. Functional and evolutionary implications of gene orthology. Nat Rev Genet (2013) 14(5):360–6. doi: 10.1038/nrg3456 PMC5877793 23552219

[37] Jensen LJ Julien P Kuhn M von Mering C Muller J Doerks T. eggNOG: automated construction and annotation of orthologous groups of genes. Nucleic Acids Res (2008) 36(Database issue):D250–4. doi: 10.1093/nar/gkm796 PMC2238944 17942413

[38] Tran NT Gao ZX Zhao HH Yi SK Chen BX Zhao YH. Transcriptome analysis and microsatellite discovery in the blunt snout bream (Megalobrama amblycephala) after challenge with aeromonas hydrophila. Fish Shellfish Immunol (2015) 45(1):72–82. doi: 10.1016/j.fsi.2015.01.034 25681750

[39] Kanehisa M Furumichi M Sato Y Ishiguro-Watanabe M Tanabe M. KEGG: integrating viruses and cellular organisms. Nucleic Acids Res (2021) 49(D1):D545–51. doi: 10.1093/nar/gkaa970 PMC7779016 33125081

[40] Sadeqzadeh E de Bock CE Thorne RF. Sleeping giants: emerging roles for the fat cadherins in health and disease. Med Res Rev (2014) 34(1):190–221. doi: 10.1002/med.21286 23720094

[41] Tiwari P Mrigwani A Kaur H Kaila P Kumar R Guptasarma P. Structural-mechanical and biochemical functions of classical cadherins at cellular junctions: A review and some hypotheses. Adv Exp Med Biol (2018) 1112:107–38. doi: 10.1007/978-981-13-3065-0_9 30637694

[42] Palomino DC Marti LC. Chemokines and immunity. Einstein (Sao Paulo) (2015) 13(3):469–73. doi: 10.1590/S1679-45082015RB3438 PMC4943798 26466066

[43] Du Y Miao W Jiang X Cao J Wang B Wang Y. The epithelial to mesenchymal transition related gene calumenin is an adverse prognostic factor of bladder cancer correlated with tumor microenvironment remodeling, gene mutation, and ferroptosis. Front Oncol (2021) 11:683951. doi: 10.3389/fonc.2021.683951 34150647 PMC8209417

[44] Ng TB Fai Cheung RC Wing Ng CC Fang EF Wong JH. A review of fish lectins. Curr Protein Pept Sci (2015) 16(4):337–51. doi: 10.2174/138920371604150429160850 25929869

[45] Mu L Yin X Wu H Han K Guo Z Ye J. MAp34 regulates the non-specific cell immunity of Monocytes/Macrophages and inhibits the lectin pathway of complement activation in a teleost fish. Front Immunol (2020) 11:1706. doi: 10.3389/fimmu.2020.01706 32903484 PMC7435015

[46] Petit J Bailey EC Wheeler RT de Oliveira CAF Forlenza M Wiegertjes GF. Studies into β-glucan recognition in fish suggests a key role for the c-type lectin pathway. Front Immunol (2019) 10:280. doi: 10.3389/fimmu.2019.00280 30863400 PMC6400144

[47] Sequeida A Maisey K Imarai M. Interleukin 4/13 receptors: An overview of genes, expression and functional role in teleost fish. Cytokine Growth Factor Rev (2017) 38:66–72. doi: 10.1016/j.cytogfr.2017.09.004 28988781

[48] Sun G Guillon E Holley SA. Integrin intra-heterodimer affinity inversely correlates with integrin activatability. Cell Rep (2021) 35(10):109230. doi: 10.1016/j.celrep.2021.109230 34107244 PMC8227800

[49] Alessandrini F Pezzè L Ciribilli Y. LAMPs: Shedding light on cancer biology. Semin Oncol (2017) 44(4):239–53. doi: 10.1053/j.seminoncol.2017.10.013 29526252

[50] Gao X Cao Q Cheng Y Zhao D Wang Z Yang H. Chronic stress promotes colitis by disturbing the gut microbiota and triggering immune system response. Proc Natl Acad Sci USA (2018) 115(13):E2960–9. doi: 10.1073/pnas.1720696115 PMC5879702 29531080

[51] Lima C Disner GR Falcão MAP Seni-Silva AC Maleski ALA Souza MM. The natterin proteins diversity: A review on phylogeny, structure, and immune function. Toxins (Basel) (2021) 13(8):538. doi: 10.3390/toxins13080538 34437409 PMC8402412

